# Gene Regulatory Network Inference from Multifactorial Perturbation Data Using both Regression and Correlation Analyses

**DOI:** 10.1371/journal.pone.0043819

**Published:** 2012-09-21

**Authors:** Jie Xiong, Tong Zhou

**Affiliations:** 1 Department of Automation, Tsinghua University, Beijing, China; 2 Department of Automation and Tsinghua National Laboratory for Information Science and Technology(TNList), Tsinghua University, Beijing, China; CRS4, Italy

## Abstract

An important problem in systems biology is to reconstruct gene regulatory networks (GRNs) from experimental data and other a priori information. The DREAM project offers some types of experimental data, such as knockout data, knockdown data, time series data, etc. Among them, multifactorial perturbation data are easier and less expensive to obtain than other types of experimental data and are thus more common in practice. In this article, a new algorithm is presented for the inference of GRNs using the DREAM4 multifactorial perturbation data. The GRN inference problem among 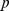 genes is decomposed into 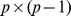 different regression problems. In each of the regression problems, the expression level of a target gene is predicted solely from the expression level of a potential regulation gene. For different potential regulation genes, different weights for a specific target gene are constructed by using the sum of squared residuals and the Pearson correlation coefficient. Then these weights are normalized to reflect effort differences of regulating distinct genes. By appropriately choosing the parameters of the power law, we constructe a 0–1 integer programming problem. By solving this problem, direct regulation genes for an arbitrary gene can be estimated. And, the normalized weight of a gene is modified, on the basis of the estimation results about the existence of direct regulations to it. These normalized and modified weights are used in queuing the possibility of the existence of a corresponding direct regulation. Computation results with the DREAM4 *In Silico* Size 100 Multifactorial subchallenge show that estimation performances of the suggested algorithm can even outperform the best team. Using the real data provided by the DREAM5 Network Inference Challenge, estimation performances can be ranked third. Furthermore, the high precision of the obtained most reliable predictions shows the suggested algorithm may be helpful in guiding biological experiment designs.

## Introduction

Reconstructing the structure of a gene regulatory network (GRN) from experimental data and other a priori information is very helpful in understanding the development, pathology and functioning of all biological organisms. Recently, with the development of high-throughput technologies, such as DNA microarrays, mass spectroscopy, etc., it is possible to reconstruct GRNs from some types of experimental data. In practice, the common data types contain knockout data, knockdown data, time series data, etc. Various models and methods have been suggested to attack this problem based on these types of experimental data, such as Boolean networks [1], Bayesian networks [2], information theory based algorithms [3], ordinary differential equation (ODE) based methods [4], etc.

Recently, the Dialogue for Reverse Engineering Assessments and Methods (DREAM) has been providing not only a set of benchmark networks extracted from actual biological networks of some most important and typical biological modules, such as *Escherichia coli* transcriptional regulatory network and *Saccharomyces cerevisiae* (yeast) transcriptional regulatory network [5], but also some types of *In Silico* gene expression data sets generated by the GeneNetWeaver tool version 2.0 [6], to motivate the systems biology community to investigate and develop structure identification methods for GRNs. In particular, the DREAM project offers an alternative type of steady-state data, i.e., multifactorial perturbation data, which are obtained by slightly perturbing all genes simultaneously so that the basal activation of all genes of the network is slightly increased or decreased simultaneously by different random amounts [5]. Multifactorial perturbation data might be regarded as expression profiles obtained from different patients [5]. Therefore, such data are easier and less expensive to be obtained than other types of experimental data and are thus more common in practice [7]. On the other hand, such data provide less information about GRNs with respect to other types of data which make the GRN identification problem more formidable [7].

Several methods have been shown to be effective in inferring the structure of a GRN through participating in the DREAM4 *In Silico* Size 100 Multifactorial subchallenge. For example, the best performer has developed GENIE3 algorithm for the inference of GRNs, which decomposes the prediction of a regulatory network among 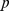 genes into 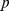 different regression problems. In each of the regression problems, the expression pattern of a target gene is predicted from the expression patterns of all the other genes, using tree-based methods [7]. The second place team tackled the problem *via* a sparse Gaussian Markov Random Field, which relates network topology with the covariance inverse generated by the gene measurements. And, the Graphical Lasso algorithm is used to compute the covariance inverse. Then, the optimal network is selected by different model selection criteria [8]. On the other hand, a GRN can be modeled as a correlation network [9], which is obtained by computing the correlation coefficient between arbitrary two genes. Surprisingly but also interestingly, this simple method was proved to be placed at the second (tie) for the DREAM4 *In Silico* Size 100 Multifactorial subchallenge. However, due to the symmetry of the correlation coefficient, the estimated correlation network topology is undirected.

Motivated by the GENIE3 algorithm, an identification algorithm is developed in this paper for GRN topology inference, based on the regression analysis and the correlation analysis. Specifically, the GRN inference problem among 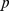 genes is decomposed into 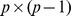 different regression problems. And, in each of the regression problems, the expression level of a target gene is predicted solely from the expression level of a potential regulation gene. For different potential regulation genes, different weights for a specific target gene are constructed. The larger the sum of squared residuals is, the weaker the direct regulatory interaction will be. And, the higher the Pearson correlation coefficient is, the stronger the rationality is for the application of the linear regression. To take both into consideration, the weight corresponding to a possible direct regulation is selected as their product. Then these weights are normalized to reflect effort differences of regulating distinct genes.

It has been observed that most large scale gene regulatory networks are sparse. Mathematically, the sparsity of a GRN may be characterized by the power law [4]. And, the in-degree distribution of a GRN can be obtained by means of the power law. In this paper, the so-called in-degree distribution means the number of genes with in-degree equal to 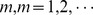. By appropriately choosing the in-degree distribution of a GRN, this paper suggest a method to utilize the sparsity quantitatively. Through constructing loss functions and incorporating power law, and solving a 0–1 integer programming problem, the direct regulation genes for an arbitrary gene can be estimated. Then, the above normalized weights can be further adjusted based on these estimated direct regulatory relationships.

In general, these weights are used to queue the possibility of the direct causal regulation. The larger the adjusted weight is, the higher the confidence is for the existence of the direct causal regulation. When a threshold is provided, this queue can lead to an estimate about the structure of a GRN. Computation results with the DREAM4 *In Silico* Size 100 Multifactorial subchallenge show that estimation performances of the suggested algorithm can even outperform the best team. Using the real data provided by the DREAM5 Network Inference Challenge, estimation performances by the proposed method can be ranked third. Furthermore, the high precision of the obtained most reliable predictions implies that the suggested algorithm may be helpful in guiding biological validation experiment designs.

The outline of this paper is as follows. At first, the structure estimation algorithm is illustrated. Afterwards, the proposed estimation method is assessed using the data sets of the DREAM4 *In Silico* Size 100 Multifactorial subchallenge and the DREAM5 Network Inference Challenge. Variations of estimation performances with respect to parameters of the suggested method will also be reported. Finally, some concluding remarks are given about the characteristics of the suggested method, as well as some future works worthy of further efforts.

## Materials and Methods

### Problem Statement

Considering a GRN with 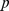 genes, it is assumed that the targeted network is a directed graph, in which each node represents a gene, and an edge directed from one gene 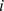 to another gene 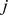 indicates that gene 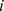 regulates the expression of gene 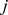 directly. The goal of gene regulatory network inference in this paper is to recover the network solely from multifactorial perturbation data. A set of multifactorial perturbation data can be obtained by first perturbing all genes simultaneously, and then measuring steady-state levels of all genes. Different data sets can be obtained by implementing different perturbations to the network [5]. At the same time, such data do not give information about the regulatory network dynamics, but about the system equilibrium once it has recovered after the perturbation [8].

Denote 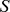 by 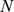 sets of multifactorial perturbation data:
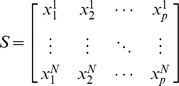
where, 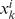 represents the steady-state levels of gene 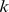 in the 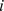-th experiment. Specifically, the problem of recovering regulatory networks is addressed as follows:

Utilizing data set 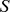, design a GRN inference algorithm and assign weights 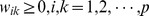. The larger the weight 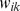 is, the higher the confidence is for the existence of the direct causal regulation from gene 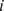 to gene 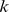.

For most of large scale networks, it has been observed that the distribution of the number of chemical elements that have direct regulatory effects on a randomly chosen chemical element, obeys approximately a power law [4], [10]–[13]. More specifically, let 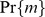 denote the probability that the number of direct regulations on a randomly chosen chemical element equals to 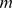, then there exist a positive number 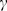 and a positive integer 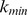, such that [4]

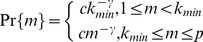
in which 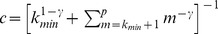. This important prior structural information is also incorporated into our estimation procedures.

### Regression Analysis

It is well known that the relevance between any two genes can be represented by the Pearson correlation coefficient [9]. But this method is non-causal. On the other hand, the GENIE3 algorithm decomposes the prediction of a regulatory network among 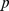 genes into 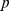 different regression problems. In each of the regression problems, the expression pattern of a target gene is predicted from the expression patterns of all the other genes, using tree-based methods [7]. Motivated by this idea, we decompose the GRN inference problem among 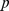 genes into 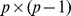 different regression problems. The novelty is as follows. In each of the regression problems, the expression level of a target gene is predicted solely from the expression level of a potential regulation gene. For different potential regulation genes, different weights for a specific target gene are constructed. That is, for a particular gene 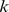 and its potential regulation gene 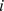, the aim of the regression analysis is to establish a function, i.e., 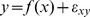. Obviously, this function reveals the causal relationship between them. Here, 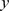 and 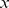 represent the steady state expression concentrations of the genes 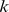 and 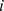, respectively. In practice, 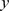 is not completely determined by 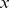, because there are many factors which may affect 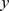. Therefore, 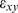 is used to represent the unknown secondary factors or/and the random errors, all of which may affect 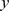. An important parameter in the regression model is the variance of 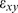, i.e., 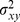. In essence, 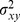 is the mean squared error when 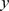 is approximated by an suitable function 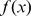 [14]. Generally, when 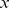 is reasonably selected as the most important factor, then the value of 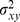 will be relatively smaller; otherwise, the value of 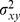 will be relatively larger. In other words, for the particular gene 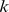, the smaller the magnitude of 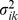 is, the larger the probability is for the existence of the direct causal regulation from gene 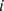 to gene 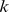.

In practice, although 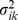 is unavailable, it can be estimated from the sum of squared residuals by using linear regression (least-squares estimation). Therefore, we can construct the weight 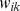 based on the above discussion. A practical network prediction is obtained by setting a threshold on the ranking of weights from the most to the less significant. In this paper, we focus on the task of constructing weights, while the question of the choice of an optimal confidence threshold, although important, will be left open.

### Weight Construction

Denote by 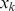 the steady-state level of gene 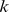. The steady-state level of gene 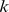 may be directly affected by all other genes expression levels. Therefore we have the following expression:
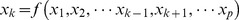
(1)


The function 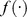 in Equation (1) not only contains lots of arguments, but also may be a non-linear function. Thus, it is hard to directly estimate the function 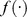. On the other hand, from the definition of the weight 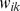, we know that 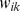 represents the probability of the direct causal regulation only from gene 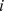 to gene 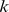. That is, when the weight 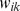 is computed, the function 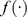 in Equation (1) can be approximated by the following expression:
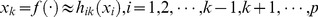
(2)


The form of the function 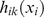, however, is not clear and might be non-linear. Hence, the linear regression technique is used to analyze the direct causal regulation from gene 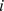 to gene 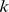. And, the function 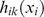 is approximated by its first order Taylor expansion, i.e.,
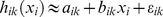
(3)where, 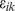 represents the approximation error or/and the measurement error.

Consequently, from Equation (2) and Equation (3), we have
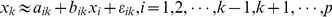
(4)The regression coefficients 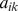 and 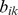 can be estimated by the least squares estimation. Let 
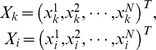
, then 
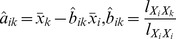
, Here, 
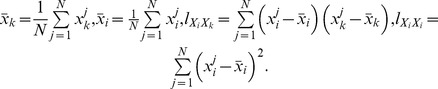
 Moreover, the sum of squared residuals 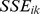 is also obtained in this process, i.e.,
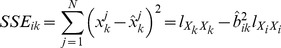
(5)where, 
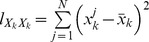
. The value of 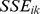 might be regarded as the capability of the direct regulatory interaction from gene 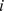 to gene 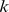. In other words, we assume that the larger the sum of squared residuals 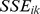 is, the weaker the direct regulatory interaction from gene 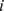 to gene 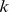 will be. For this reason, the constructed weight should utilize this characteristic provided by 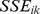.

On the other hand, for arbitrary two data sets 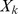 and 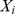, not matter whether there exists the linear correlation between them, the sum of squared residuals 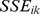 can always be obtained by Equation (5). However, if there does not exist the linear correlation between them, the application of the linear regression is meaningless. To test whether the data sets 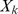 and 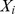 are linear correlation, the correlation coefficient is the most frequently used test statistic. The expression for the correlation coefficient 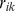 is as follows:
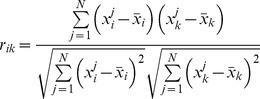
(6)


According to the discussion above, it is clear that the larger the sum of squared residuals 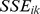 is, the weaker the direct regulatory interaction from gene 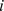 to gene 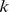 will be. And, the larger the Pearson correlation coefficient 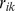 is, the stronger the rationality is for the application of the linear regression on data sets 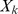 and 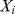. To take both of them into consideration, a weight 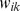 corresponding to a possible direct regulation from gene 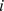 to gene 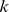 is constructed as follows:
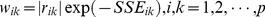
(7)For the particular gene 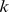, the larger the magnitude of 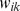 is, the larger the confidence is that gene 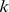 is directly regulated by gene 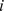.

### Weight Normalization

It is important to note that in GRN topology inferences the larger the value of 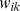 is, the larger the probability is for the existence of a direct regulation from gene 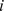 to gene 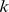. Define a 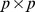 dimensional matrix 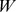 with its 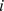-th row 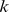-th column element being the estimate of 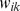 when 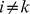 and its diagonal element being zero, and denote its 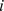-th column vector by 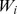. And then, it is clear that this matrix contains information about the probability of the existence of a direct regulation between any two different genes in a GRN. However, to infer the structure of a GRN from this matrix, an important fact must be taken into account. That is, in a GRN, some genes may be easily regulated by other genes, while regulations on some other genes may need more efforts [15]–[17]. This implies that direct regulations to different genes may lead to weights of different magnitude orders. Therefore, in order to obtain a good estimate from the matrix 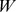 about the topology of a GRN, an appropriate normalization is still required for the estimated 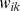
s among different genes.

In [17], it is suggested to use the 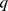-norm of the vector 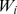 and the geometric average of its non-zero elements to achieve the normalization. More specifically, when 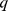 is adopted as 3.5, the structure inference performance is improved the most. Therefore, in this paper, it is suggested to also use the 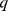-norm of the vector 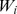 to achieve this normalization, that is, 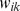 is replaced by
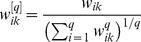
(8)It is worthwhile to note that this normalization does not change the diagonal elements. For presentation conciseness, the normalized matrix 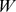 using the vector 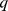-norm is denoted by 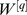 in the rest of this paper.

The normalization is firstly proposed in [17], in which the weight is represented by the RELV (relative expression level variation). The goal of the normalization is to guarantee that the weights for different genes hold the same magnitude order. For a GRN, in the last ranking list of 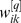, if the magnitude is larger, the corresponding transcription regulation will be established in a larger probability.

### In-degree Estimation and Weight Magnitude Modification

To compute the weight 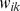, the multivariate function 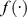 is approximated by a univariate function 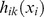, which implies that the in-degree for an arbitrary gene 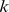 is assumed as one. Thus, the constructed weights do not employ the information about the combinatorial regulation to a gene. In this subsection, we try to estimate the in-degrees of genes in a GNR to utilize the information about the combinatorial regulation.

It is clear that the value of 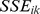 represents the capability of the direct regulatory interaction from gene 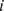 to gene 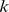, that is, the smaller the sum of squared residuals 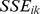 is, the stronger the direct regulatory interaction from gene 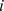 to gene 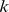 will be. Sort the sum of squared residuals of gene 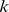 in a non-decreasing order, and denote the sorted results as follows:
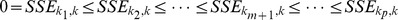
In this ranking 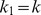, so it is assumed that the top 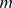 genes from gene 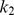 to gene 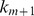 have great chance to combinatorially regulate gene 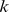. Therefore, the multivariate function 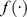 can be approximated by a 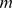-variable function in such case, i.e.:
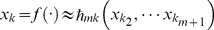
(9)The form of the function 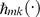, however, is also not clear and might be non-linear. Hence, the linear regression technique is used again. Applying the first order multiple Taylor expansion to the function 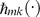, we have
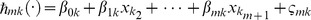
where, 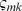 represents the approximation error or/and the measurement error.

Using the least squares again, not only the regression coefficients 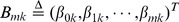, but also the sum of squared residuals 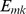 and the sum of deviation squares 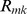 can be estimated. Let
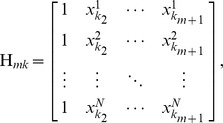
then,
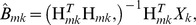
and,
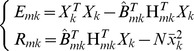
(10)


Define a loss function 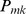 as follows:
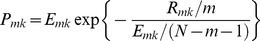
(11)Here, the value of the sum of squared residuals 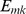 represents the capability of a direct combinatorial regulation from genes numbered 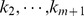 to gene 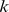. Obviously, it can be thought that the smaller the sum of squared residuals 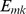 is, the stronger the direct combinatorial regulation interaction will be. And, 
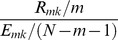
 is the test statistic. The larger the test statistic is, the stronger the rationality is for the application of the multiple linear regression analysis. Therefore, to take both into consideration, the loss function 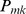 is defined as Equation (11). And, it can be presumed that the smaller the value of 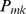 is, the higher the probability is for the establishment of a direct combinatorial regulation from genes numbered 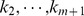 to gene 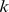.

To estimate the in-degree for a specific gene 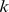 optimally, one can search 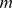 from 1 to 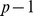 to find the minimum of the loss function 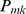 at 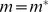. In such case, the optimal in-degree for the specific gene 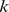 is 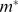 and genes numbered 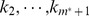 are most likely to have a direct regulation effect on gene 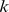. However, to estimate the in-degree for every gene in a GRN optimally, the structural characteristics of GRNs should be taken into consideration, that is, the power low could be taken into consideration. let 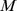
(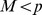) and 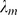 denote respectively the maximum in-degree of a GRN and the number of genes with its in-degree equalling to 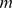. Then, from the power law, it is clear that 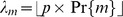. Since each gene has a unique in-degree, we can utilize the following 0–1 integer optimization to estimate the in-degree for every gene optimally.
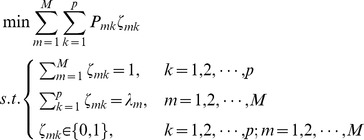
(12)


Problem (12) can be solved by using a linear programming-based branch-and-bound algorithm [18], [19], and its optimal estimates can be denoted by 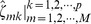. For gene 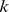, if 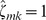, with 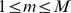, then, from the above problem description, it is clear that the optimal estimate for the in-degree of this gene is 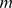, and genes numbered 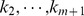 are most likely to have a direct regulation effect on this gene.

In GRN topology estimation, another important thing worthy of considering is that genes estimated to have a direct regulation should correspond to a weight with a magnitude greater than those estimated not to have a direct regulation [20], [21]. To achieve this purpose, the following adjustment is suggested in this paper. Define 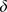 as
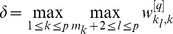
(13)With this value, the normalized weights for an arbitrary gene 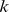 are modified as follows,
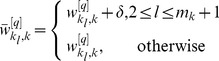
(14)Here, for each gene 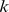, 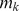 is determined by the solution of Problem (12).

Denote by 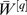 the 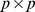 dimensional matrix with its 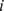-th row 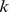-th column element being 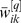. Elements of this matrix are directly used to infer the structure of a GRN. The bigger the 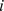-th row 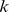-th element is, the higher the probability is that gene 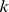 is directly regulated by gene 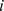.

It should be stressed here that the effectiveness of the in-degree estimation depends on the veracity of the prior structural information. In this paper, the sparsity of a GRN is characterized by the power law. Therefore, the number of genes, whose in-degree are equal to 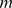, can be represented as 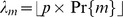. Here, 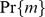 is the so-called power law. That is, the solution of Problem (12) depends on the parameters of the power law. If the in-degree distribution of a GRN is pertinent and appropriate, the effectiveness of this step may be positive. Otherwise the performance may deteriorate. The results from Table 1,2,3 in the following section may support the argument.

**Table 1:** Performances Only for Weight Normalization.

		Net1	Net2	Net3	Net4	Net5	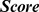
Best Team	AUROC	0.745	0.733	0.775	0.791	0.798	37.428
	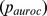	(3.334e-18)	(1.076e-28)	(9.705e-34)	(6.736e-33)	(1.912e-34)	
	AUPR	0.154	0.155	0.231	0.208	0.197	
	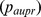	(3.309e-34)	(7.897e-54)	(1.791e-54)	(5.489e-47)	(4.563e-44)	
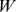	AUROC	0.6899	0.6485	0.7081	0.6998	0.6655	16.4038
	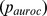	(1.4643e-12)	(1.6105e-13)	(1.2922e-20)	(5.6590e-18)	(6.6749e-13)	
	AUPR	0.0711	0.0893	0.1230	0.0938	0.0532	
	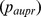	(5.3481e-14)	(1.9060e-24)	(3.2720e-27)	(6.6316e-19)	(3.5962e-09)	
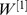	AUROC	0.7524	0.7097	0.7694	0.7590	0.7651	35.7303
	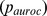	(5.3124e-19)	(5.3557e-24)	(1.4810e-32)	(2.9514e-27)	(3.7157e-28)	
	AUPR	0.1464	0.1673	0.2212	0.2046	0.1944	
	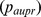	(2.2398e-32)	(1.7894e-59)	(4.0881e-52)	(3.0186e-46)	(2.1768e-43)	
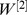	AUROC	0.7614	0.7149	0.7690	0.7697	0.7691	38.1179
	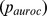	(5.1100e-20)	(5.0426e-25)	(1.8088e-32)	(4.0920e-29)	(6.5978e-29)	
	AUPR	0.1626	0.1697	0.2283	0.2229	0.2271	
	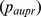	(2.4910e-36)	(1.4524e-60)	(6.1040e-54)	(9.2038e-51)	(2.5905e-51)	
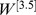	AUROC	0.7641	0.7172	0.7660	0.7762	0.7693	38.4670
	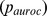	(2.5068e-20)	(1.7462e-25)	(8.0330e-32)	(2.7481e-30)	(6.3238e-29)	
	AUPR	0.1673	0.1612	0.2273	0.2271	0.2448	
	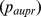	(1.7753e-37)	(1.5096e-56)	(1.1603e-53)	(8.4634e-52)	(1.3305e-55)	

**Table 2:** Performances with the optimal 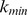 and 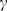.

		Net1	Net2	Net3	Net4	Net5	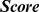
Best Team	AUROC	0.745	**0.733**	0.775	**0.791**	**0.798**	37.428
	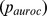	(3.334e-18)	(1.076e-28)	(9.705e-34)	(6.736e-33)	(1.912e-34)	
	AUPR	0.154	0.155	0.231	0.208	0.197	
	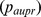	(3.309e-34)	(7.897e-54)	(1.791e-54)	(5.489e-47)	(4.563e-44)	
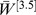	AUROC	**0.7642**	0.7173	**0.7865**	0.7764	0.7693	39.9465
	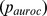	(2.4413e-20)	(1.6671e-25)	(2.2385e-36)	(2.5303e-30)	(6.0610e-29)	
	AUPR	**0.1799**	**0.1648**	**0.2341**	**0.2326**	**0.2540**	
	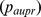	(1.4115e-40)	(2.7231e-58)	(2.1883e-55)	(3.7182e-53)	(7.8435e-58)	
	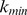	1	1	3	2	1	
	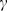	3.3	3.3	1	3.7	5.0	

**Table 3:** Performances with typical 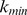 and 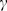.

		Net1	Net2	Net3	Net4	Net5	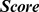
Best Team	AUROC	0.745	0.733	0.775	0.791	0.798	37.428
	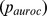	(3.334e-18)	(1.076e-28)	(9.705e-34)	(6.736e-33)	(1.912e-34)	
	AUPR	0.154	0.155	0.231	0.208	0.197	
	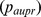	(3.309e-34)	(7.897e-54)	(1.791e-54)	(5.489e-47)	(4.563e-44)	
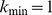 , 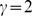	AUROC	0.7634	0.7165	0.7691	0.7752	0.7687	37.4489
	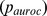	(3.0972e-20)	(2.4137e-25)	(1.8088e-32)	(4.1501e-30)	(7.8172e-29)	
	AUPR	0.1710	0.1564	0.2290	0.2047	0.2287	
	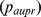	(2.2195e-38)	(2.4170e-54)	(4.2999e-54)	(3.0186e-46)	(1.0608e-51)	
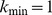 , 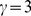	AUROC	0.7641	0.7171	0.7674	0.7759	0.7694	38.1914
	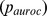	(2.5068e-20)	(1.9156e-25)	(4.0136e-32)	(3.1102e-30)	(5.8091e-29)	
	AUPR	0.1720	0.1598	0.2263	0.2189	0.2394	
	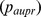	(1.2653e-38)	(6.1630e-56)	(1.9625e-53)	(8.9338e-50)	(2.5610e-54)	
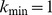 , 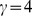	AUROC	0.7641	0.7173	0.7670	0.7762	0.7692	38.8123
	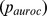	(2.5068e-20)	(1.6671e-25)	(4.8953e-32)	(2.8639e-30)	(6.3238e-29)	
	AUPR	0.1722	0.1646	0.2378	0.2173	0.2455	
	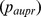	(1.1308e-38)	(3.7733e-58)	(2.5223e-56)	(2.2175e-49)	(8.5142e-56)	
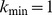 , 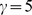	AUROC	0.7642	0.7173	0.7660	0.7765	0.7693	39.0655
	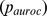	(2.5068e-20)	(1.7462e-25)	(8.0330e-32)	(2.5303e-30)	(6.0610e-29)	
	AUPR	0.1716	0.1632	0.2396	0.2212	0.2540	
	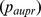	(1.5842e-38)	(1.5489e-57)	(8.8169e-57)	(2.4181e-50)	(7.8435e-58)	
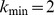 , 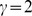	AUROC	0.7609	0.7152	0.7714	0.7756	0.7685	37.9334
	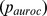	(5.8275e-20)	(4.3935e-25)	(5.7087e-33)	(3.6677e-30)	(8.8762e-29)	
	AUPR	0.1639	0.1536	0.2349	0.2216	0.2380	
	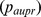	(1.1998e-36)	(4.9060e-53)	(1.3716e-55)	(2.0391e-50)	(5.9144e-54)	
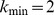 , 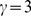	AUROC	0.7630	0.7166	0.7688	0.7758	0.7687	38.2446
	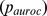	(3.3525e-20)	(2.3048e-25)	(1.9987e-32)	(3.3776e-30)	(7.8172e-29)	
	AUPR	0.1634	0.1574	0.2334	0.2271	0.2397	
	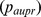	(1.5022e-36)	(9.1641e-55)	(3.2932e-55)	(8.4634e-52)	(2.2905e-54)	
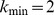 , 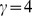	AUROC	0.7636	0.7170	0.7687	0.7762	0.7690	38.4010
	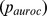	(2.9378e-20)	(2.0063e-25)	(2.2085e-32)	(2.7481e-30)	(6.8837e-29)	
	AUPR	0.1652	0.1599	0.2312	0.2270	0.2410	
	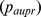	(5.7785e-37)	(6.1630e-56)	(1.1225e-54)	(8.9583e-52)	(1.1089e-54)	
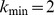 , 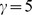	AUROC	0.7637	0.7171	0.7687	0.7762	0.7692	38.3533
	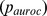	(2.7865e-20)	(1.8289e-25)	(2.2085e-32)	(2.7481e-30)	(6.5978e-29)	
	AUPR	0.1668	0.1612	0.2284	0.2216	0.2428	
	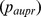	(2.2228e-37)	(1.3546e-56)	(5.7578e-54)	(2.0391e-50)	(4.0615e-55)	
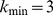 , 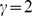	AUROC	0.7586	0.7167	0.7730	0.7745	0.7685	38.0248
	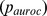	(1.0644e-19)	(2.2007e-25)	(2.5460e-33)	(5.5346e-30)	(8.8762e-29)	
	AUPR	0.1626	0.1567	0.2327	0.2231	0.2370	
	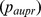	(2.4910e-36)	(1.9486e-54)	(4.6750e-55)	(8.2152e-51)	(1.0333e-53)	
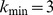 , 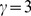	AUROC	0.7625	0.7170	0.7710	0.7750	0.7690	38.3306
	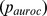	(3.8251e-20)	(1.9156e-25)	(6.6388e-33)	(4.5061e-30)	(6.8837e-29)	
	AUPR	0.1682	0.1577	0.2340	0.2231	0.2396	
	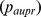	(1.0706e-37)	(6.6312e-55)	(2.3199e-55)	(8.2152e-51)	(2.4220e-54)	
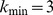 , 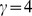	AUROC	0.7631	0.7175	0.7702	0.7759	0.7689	38.7893
	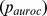	(3.2651e-20)	(1.5916e-25)	(9.9210e-33)	(3.2412e-30)	(7.1819e-29)	
	AUPR	0.1682	0.1624	0.2369	0.2274	0.2414	
	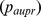	(1.0706e-37)	(3.6898e-57)	(4.2661e-56)	(7.1370e-52)	(8.8708e-55)	
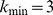 , 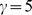	AUROC	0.7634	0.7165	0.7693	0.7760	0.7686	38.4687
	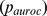	(3.0972e-20)	(2.4137e-25)	(1.6368e-32)	(3.1102e-30)	(8.5083e-29)	
	AUPR	0.1696	0.1586	0.2351	0.2270	0.2381	
	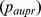	(4.8746e-38)	(2.2538e-55)	(1.2204e-55)	(8.9583e-52)	(5.2898e-54)	

### Estimation Algorithm

In summary, on the basis of the regression analysis and the correlation analysis, the algorithm suggested in this paper for identifying direct regulations of a GRN consists of the following steps.

1. Compute the weight matrix 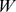 according to Equations (5), Equations (6) and Equations (7).
2. Normalize the weight matrix 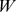 according to Equations (8).
3. Choose appropriate values for 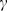, 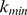 and 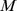, and solve the Problem (12), and modify the matrices 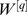 according to Equations (13) and (14). (This is an optional step, not necessary.)

Using elements of these matrices 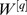 (or 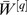), queue possibilities of the existence of a direct regulation from the gene with the same number of the row to the gene with the same number of the column. The bigger the element is, the higher the confidence is for the existence of the direct causal regulation.

## Results and Discussion

### Data Sets and Assessment Metrics

To illustrate the effectiveness of the developed inference algorithm, tests are firstly performed on the DREAM4 *In Silico* Size100 Multifactorial subchallenge, which are designed to assess performances of an identification method for the structure of a large scale GRN [22]. They respectively contain 5 different benchmark networks with 100 genes which are obtained through extracting some important and typical modules from actual biological networks of *E. coli* and *S. cerevisiae*. Auto-regulatory interactions are removed, that is, there are no self-interactions in the *in silico* networks. For each network, 100 sets of multifactorial perturbation data are supplied.

Predictions are compared with the actual structure of the networks by the DREAM project organizers using the following two different metrics in topology prediction accuracy evaluations.

- AUPR: The area under the precision-recall curve;
- AUROC: The area under the receiver operating characteristic curve.

Moreover, for every network, the 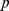-values of the AUPR and AUROC measures, which indicate the probability that random predictions would have the same or better performances, are computed, which are respectively denoted by 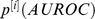 and 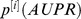, 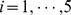. Based on these 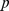-values, a final score is calculated as 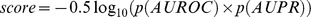. A larger score indicates a better performance of the adopted inference algorithm. Here, 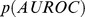 and 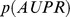 are defined as follows.
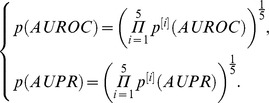
Similarly, we can define a specification for each network as 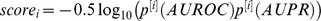, 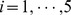. Based on the above discussion, we know
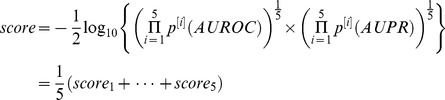



More detailed explanations can be found in [22] or on the web site of the DREAM project at http://wiki.c2b2.columbia.edu/dream/. Moreover, to evaluate performance on real data, tests are also performed on the DREAM5 Network Inference Challenge. Finally, the computation time needed by the suggested method is discussed.

### Prediction Performances of 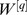


To evaluate the prediction accuracy of 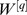, 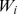 is normalized by using some typical vector norms, such as the 1-norm and the Euclidean norm. Moreover, it is reported that when 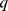 is adopted as 3.5, the structure inference performance is improved significantly [17]. Thus, each column of 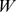 is also normalized by using the 3.5-norm. The corresponding results are given in Table 1. Also, the Performance of 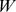 is include in Table 1.

To compare prediction performances with the best team, the corresponding specifications are also included in Table 1, obtained directly from the web site of the DREAM project. Their digit lengthes are different from the other results that are obtained through actual computations. In addition, the corresponding 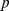-value for each specification is given in parentheses. In the last column of Table 1, the obtained scores are also given for each method. From Table 1, it is clear that by the normalization step the structure inference performance is improved remarkably. Specifically, when 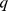 is chosen as 2 and 3.5, their final scores even outperform the best team's final score.

**Figure 1:**
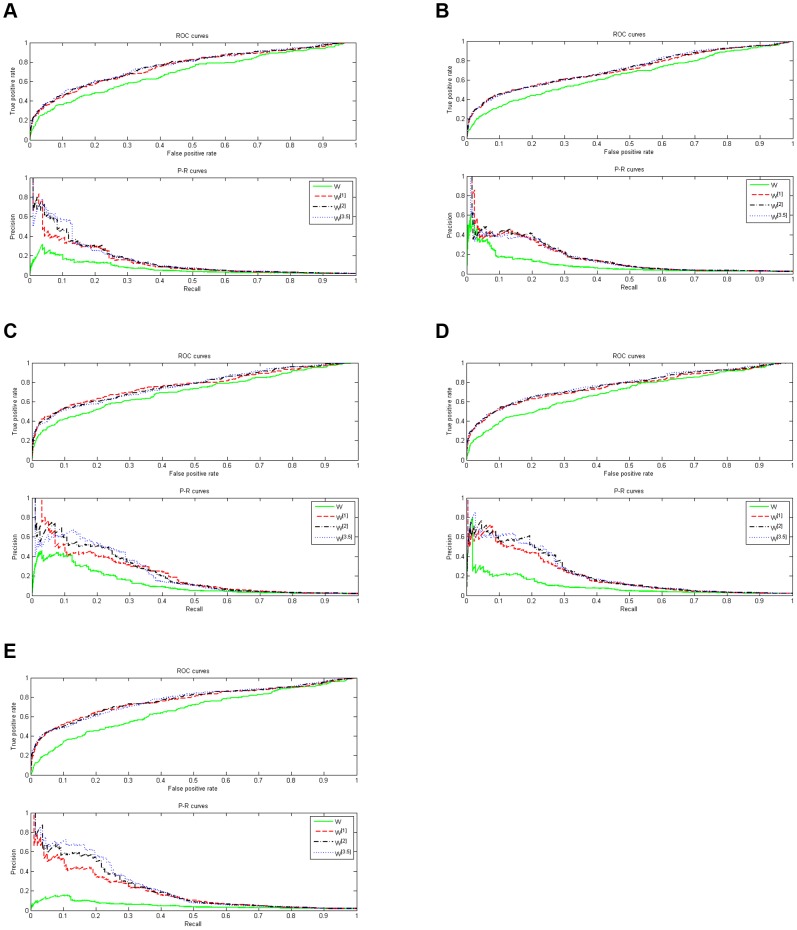
ROC and PR curves of 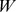
 **,** 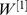 **,** 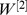 **and** 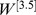 **.**

The final score is a pretty important specification in inferring the structure of GRNs, while the precision specification can not be revealed by the final score. In topology estimations, highly confident predictions can become a good guidance to biological experiment designs [22]. However, these predictions will be helpful only if their precisions are sufficiently high. This requires that a desirable estimation algorithm should have a PR (precision-recall) curve starting from the left upper corner, and decreasing monotonically and slowly with the increment of the recall rate. The ROC curve and PR curve of each network according to 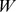, 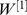, 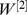 and 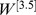 are represented in Figure 1.

From Figure 1, we can draw some conclusions as follows. The AUPR and AUROC measures of 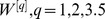, are improved much more by the normalization step compared with these measures of 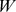. What's more, when the weight matrix is adopted as 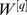, most of the PR curves start from the left upper corner. Specifically, when 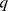 is chosen as 1 and 3.5, the precision specification is pretty well for all the five networks. And, when the weight matrix is adopted as 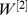, except the network 4, the PR curves start from the left upper corner for all other networks. This high precision implies that the suggested algorithm may be helpful in guiding biological validation experiment designs.

**Figure 2:**
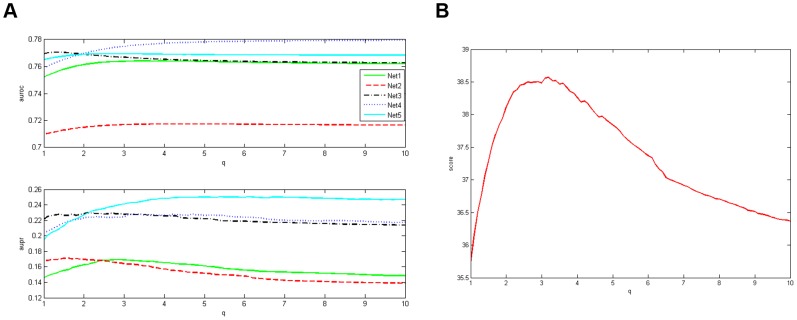
Prediction results of 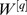. Left: Variations of the AUPR and AUROC measures with *q*; Right: Variations of the score with *q*.

To investigate how the AUPR and AUROC measures and the final score of 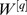 are influenced by 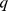, 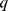 is searched over the interval 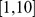 through an equally spaced sampling with 90 samples. The corresponding results are given in Figure 2.

The results in Figure 2(a) suggest that when 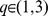, the AUROC measure for each network maintains growth along with the increase of 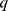. And when 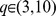, the AUROC measure for each network nearly remains unchanged. On the other hand, for the networks 2,3,4, when 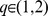, the AUPR measure maintains growth; and when 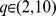, the AUPR measure slowly falls. The situation for the network 1 is similar, while the inflexion point is about 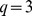. For the network 5, when 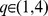, the AUPR measure maintains growth; then this measure nearly remains unchanged. As for the final score, when 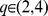, it is more than 38. And, the results in Figure 2(b) confirm again that when 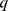 is adopted as 3.5, the structure inference performance is improved the most.

### Prediction Performances of 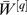


In the previous subsection, it is clear that prediction performances are improved by the normalization step, especially when the weight matrix is adopted as 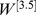. In this subsection, the prediction performances of 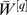 is under investigation. For convenience, 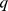 is adopted as 3.5 in this subsection.

To investigate influences of power low parameters on the prediction accuracy of the estimation algorithm, optimal values are searched for both 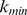 and 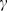. Particularly, for every network, the optimal 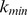 is searched over the set 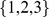, and the optimal 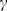 is over the interval 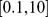 through an equally spaced sampling with 100 samples. In this optimization, the desirable 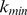 and 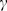 are selected to be the sample that maximizes the 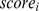 specification, 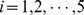. The corresponding results are given in Table 2.

Taking the exponential decay of power law into account, 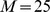 is utilized in these estimations. To compare prediction performances with the best team, the corresponding specifications are also included in Table 2, obtained directly from the web site of the DREAM project. The best values of the AUROC and the AUPR specifications for each network are written in boldface. In addition, the corresponding 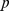-value for each specification is given in parentheses. In the last column of Table 2, the obtained scores are also given for each method. Furthermore, the optimal 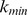 and 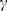 for each network are given in the last two lines. From results of Table 2, it is clear that compared with the method adopted by the best team, although there are networks with which the AUROC specification of the suggested method is slightly worse, its AUPR specification is much better than the best team for every network. Therefore, the final score for the suggested method is greater than the best team.

It is worthwhile to note that in actual applications, the optimal 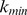 and 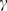 are usually not available. On the other hand, it is currently well known that for most biology systems, the parameter 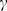 belongs to the interval 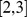 [23]. To test practical effectiveness of the suggested method, its estimation performances with the power law parameters taking some typical values, i.e., 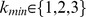 and 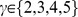 have been studied. The corresponding results are given in Table 3.

For each case, the AUROC and the AUPR specifications with the corresponding 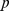-value written in parentheses are presented. And, in the last column of Table 3, the obtained scores are given for each case. In addition, similarly to Table 2, the prediction specifications of the best team are also included in Table 3. It is obvious that the performance of this step is affected by the parameters of the power law. Although estimation performance deteriorates slightly when 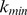 and 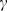 deviate from their optimal values, it is still better than the available methods.

**Figure 3:**
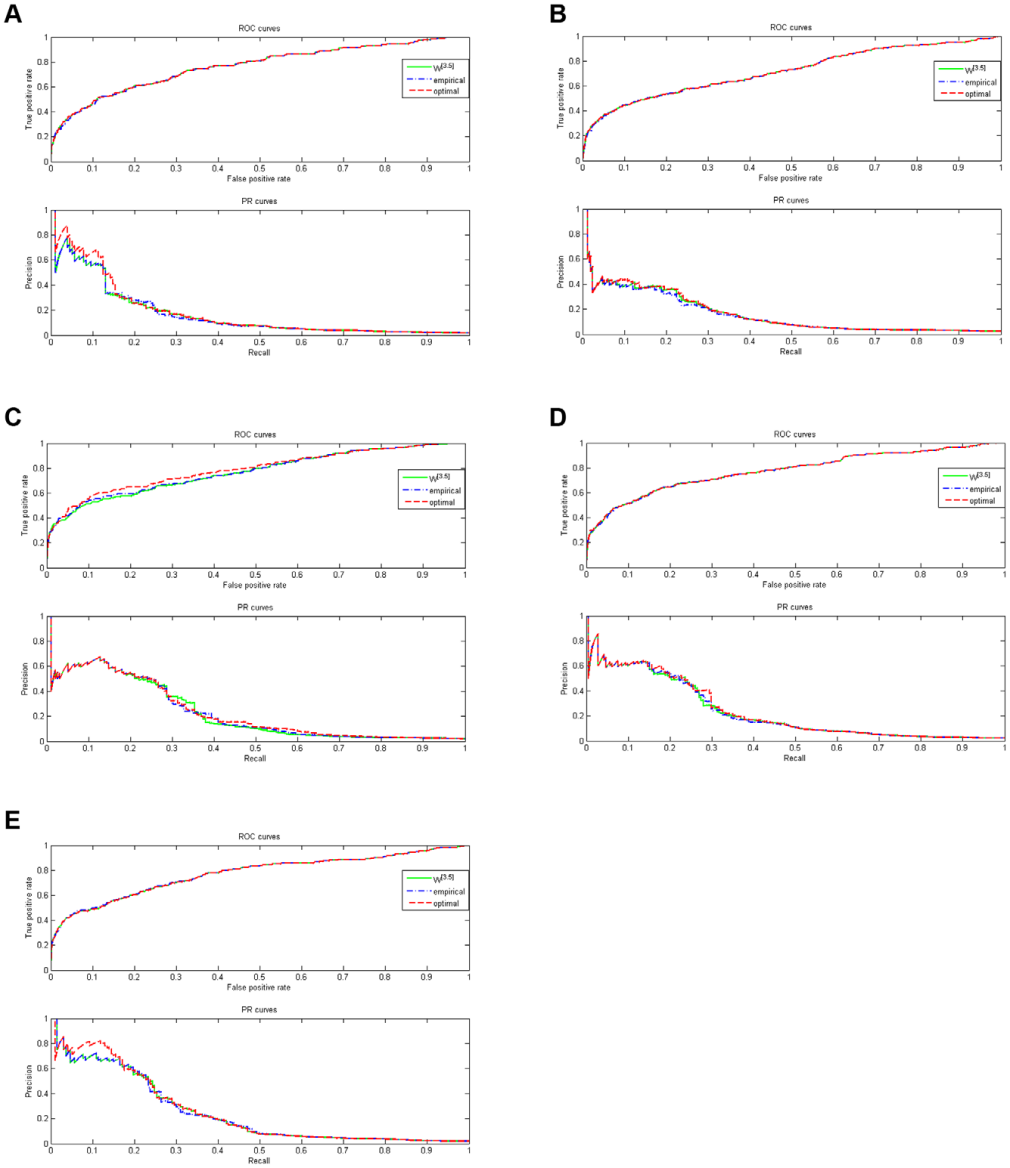
ROC and PR curves of 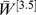.

The ROC curve and PR curve of each network with empirical and optimal power law parameters are presented in Figure 3. Here, the empirical power law parameter means that 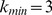 and 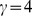 for every network.

**Figure 4:**
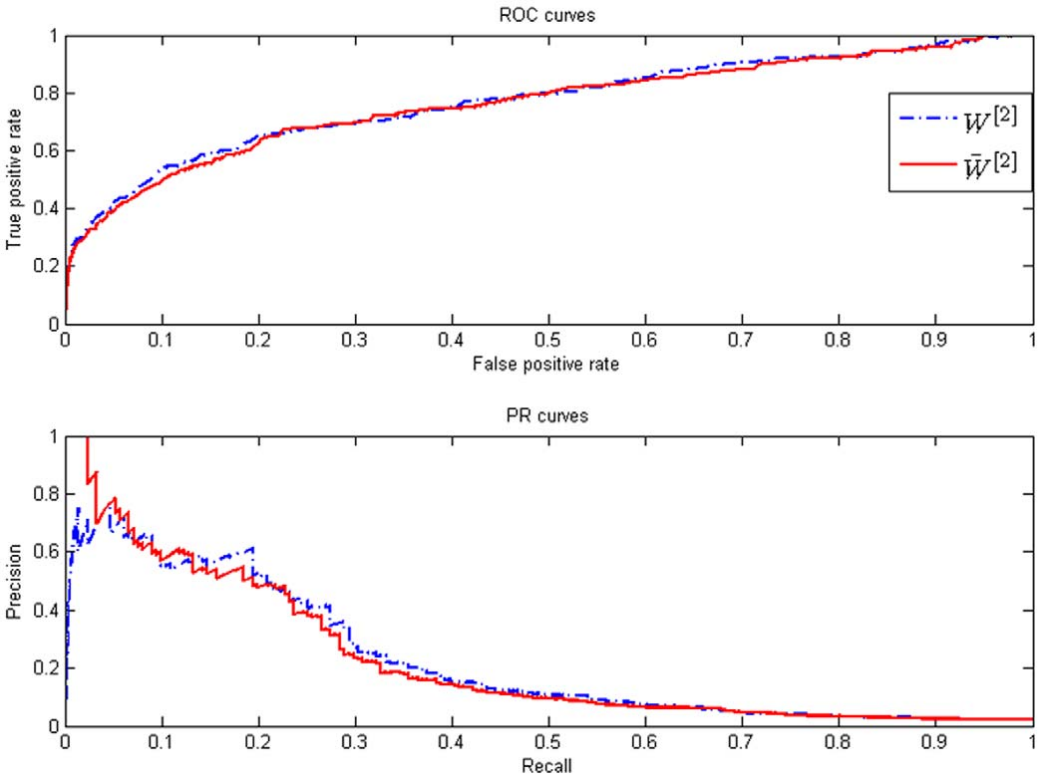
Effect for the third step.

Figure 3 show that the precision specification is also very well, when the weight matrix is adopted as 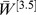. More importantly, the third step of the proposed method may guarantee that the PR curve starts from the left upper corner. This phenomenon is verified by Figure 4. Figure 4 contains two PR curves. The one is Net4 without the third step, while the other is also Net4 when its weight matrix is adopted as 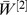. It is clear that the PR curve of 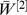 starts from the left upper corner. This feature is a good guidance to biological experiment designs.

Most large scale networks may have the sparse property, which may be approximated by the power law. The developed algorithm has quantitatively employed this property by constructing a 0–1 integer programming problem. Consequently, direct regulation genes for an arbitrary gene can be (sub)optimally estimated. Furthermore, this information is incorporated into the developed algorithm by the manipulation of Equations (13) and (14). That is the reason why the propose method has a property of high confident predictions. On the other hand, there are some potential risks when the third step is used. Specifically, when the distribution of in-degree is not accurate, the prediction accuracy of 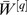 may deteriorate with respect to 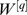. For example, when 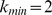 and 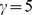, the final score of 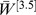 is less than 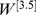. Therefore, it is suggested that when the in-degree distribution is unreliable or unavailable in practice, the operations of the third step should be used with caution.

### Performances on the DREAM5 Network Inference Challenge

To evaluate the performance on real data, tests are performed on the DREAM5 Network Inference Challenge. Here, all of gene expression data offered by the DREAM5 organizers are regarded as multifactorial perturbation data. To better reconstruct the real GRNs, some special improvements are taken into consideration. First, the networks in the DREAM5 Network Inference Challenge are much more complicated than those in the DREAM4. The function 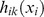 may not be properly approximated by its first order Taylor expansion. In general, if the order of the Taylor series is high enough, 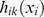 will be obtained precisely. However, this treatment may bring some adverse impacts. Especially, when 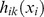 is approximated by its fourth order Taylor expansion, the matrix inversion operation will be infeasible when the least squares estimation is used. Therefore, we use the third order Taylor expansion to approximate it, i.e.,
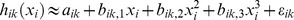
With the help of the Least Squares, the coefficients in above equation and the sum of squared residuals 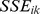 can be obtained. Second, consider two genes 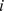 and 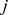. Assume that gene 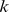 regulates gene 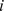 and gene 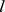 has no direct effect on gene 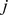. And, suppose 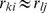 and 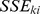 is slightly smaller than 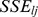. In this case, 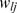 may be very close to 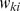 in the weight ranking list. To overcome this drawback, the factor 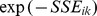 in Equation (7) is replaced by 
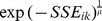
. Similarly, the factor 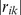 in Equation (7) is replaced by 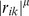. For example, suppose 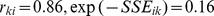 and 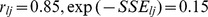, the gap between 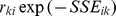 and 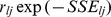 is 0.0101. While the gap between 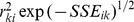 and 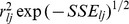 is 0.0160; and the gap between 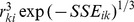 and 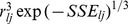 is 0.0190. In general, the value of 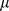 is larger than 1, but it can not be too large, to avoid 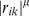 tending to 0. Based on our computational experience, when 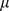 is set as 4, the performance is improved significantly. Therefore, Equation (7) is replaced by the following expression:
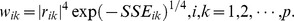



Due to the reason that the in-degree distribution is unreliable (unavailable), the operations of the third step are canceled. The prediction performances of 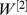 are shown in Table 4.

**Table 4:** Performances on the DREAM5 Network Inference Challenge.

		Net1	Net3	Net4	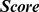
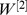	AUROC	0.7231	0.5469	0.5049	32.9093
	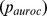	(2.2891e-10)	(0.9996)	(0.9998)	
	AUPR	0.3438	0.0595	0.0189	
	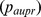	(2.2209e-185)	(7.0052e-4)	(0.9840)	

The final score in Table 4 is better than the third team. Further more, the improved method is also tested on the DREAM4 *In Silico* Size100 Multifactorial subchallenge. The final performances are represented in Table 5, and the estimation performances of the improved algorithm significantly outperform the best team. These results shows that our improved method may be competent to infer gene regulatory networks.

**Table 5:** Performances on the DREAM4 Multifactorial subchallenge using improved method.

		Net1	Net2	Net3	Net4	Net5	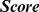
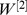	AUROC	0.7510	0.7416	0.7995	0.7865	0.8071	42.8862
	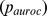	(7.6122e-19)	(1.2736e-30)	(2.1254e-39)	(3.6995e-32)	(2.7275e-36)	
	AUPR	0.1740	0.1646	0.2524	0.2472	0.2825	
	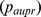	(4.1120e-39)	(3.7733e-58)	(4.7196e-60)	(9.2795e-57)	(9.7252e-65)	

### Computation Time

In this section, the computational complexity of the proposed method is discussed. It is well known that integer programming is an NP-complete problem and there is no known polynomial-time algorithm to solve it [18], [19]. Therefore, we only discuss the computational complexity of the first two steps. The main calculating module is the least squares estimator. More precisely, this estimator involves a large matrix multiplication operation, for instance 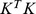. Here,
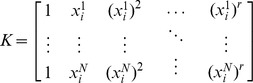
where, 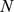 represents the experiment number, and 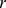 represents the order of the Taylor series. Therefore, for a particular network including 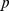 genes, in which the number of transcription factors is 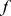, the computational complexity of the proposed method is 
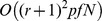
. In general, 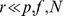, that is, the computational complexity is 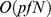.

Using the first order Taylor expansion, the computation time for each network in the DREAM4 *In Silico* Size100 Multifactorial subchallenge is respectively: 0.1047 s, 0.1054 s, 0.1042 s, 0.1052 s, and 0.1046 s. While, using the third order Taylor expansion the computation time is respectively: 0.7725 s, 0.7285 s, 0.7338 s, 0.7281 s, and 0.7272 s. For the DREAM5 Network Inference Challenge, the computation time is respectively: 65.5030 s, 378.9817 s, and 295.2905 s.

The computation is performed on a PC with Inter(R) Core (TM) i5-2400 CPU, 4 GB RAM, and Matlab 2008a.

## Concluding Remarks

In this paper, an algorithm is developed for the GRN topology inference from steady state multifactorial perturbation data. The GRN inference problem among 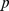 genes is decomposed into 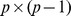 different regression problems. In each of the regression problems, the expression level of a target gene is predicted solely from the expression level of a potential regulation gene. For different potential regulation genes, different weights for a specific target gene are constructed by using the sum of squared residuals and the Pearson correlation coefficient. The larger the sum of squared residuals is, the weaker the direct regulatory interaction will be. And, the higher the Pearson correlation coefficient is, the stronger the rationality is for the application of the regression analysis. Then, the constructed weight of a gene is normalized. To employ the network sparse property quantitatively, a 0–1 integer programming problem is constructed. By solving this problem, direct regulation genes for an arbitrary gene can be estimated. Lastly, the normalized weight of a gene is modified, on the basis of the estimation results about the existence of direct regulations to it. These normalized and modified weights are used in queuing the possibility of the existence of a corresponding direct regulation.

Computational results with the DREAM4 *In Silico* Size100 Multifactorial subchallenge show that this method can outperform the available method, particularly in improving the AUPR specifications. Using the real data provided by the DREAM5 Network Inference Challenge, estimation performances can be ranked third. In addition, if the veracity of the prior structural information is certifiable, the third step of this method not only improve the final score but also could guarantee the PR curve starts from the left upper corner, which may be helpful in guiding designs of a biological validation experiment.

Although the computational results are promising, many important issues still need further efforts. Among them, how to utilize the experimental data to obtain the in-degree distribution of a GRN is currently under investigations.

## Supplementary Information

The Matlab files for this method will be offered upon request. Please contact the following email address: xiongj08@mails.tsinghua.edu.cn.
